# Dosimetric comparison of M6 CyberKnife plans optimized with Precision and RayStation 12A treatment planning systems

**DOI:** 10.1002/acm2.14585

**Published:** 2024-12-19

**Authors:** Maude Gondré, Véronique Vallet, Jean Bourhis, François Bochud, Raphael Moeckli

**Affiliations:** ^1^ Institute of Radiation Physics Lausanne University Hospital and Lausanne University Lausanne Switzerland; ^2^ Radio‐Oncology Department Lausanne University Hospital and Lausanne University Lausanne Switzerland

**Keywords:** CyberKnife, optimization, treatment planning system

## Abstract

**Purpose:**

Treatment planning for CyberKnife (CK) (Accuray, USA) can be performed with Precision (Accuray, USA) or RayStation (RS) (RaySearch Laboratories, Sweden) treatment planning systems (TPS). RaySearch recently released a new version of the CK module in RS 12A. The objective of the study was to compare plan quality between RS 12A and Precision.

**Methods:**

Fifty nine plans were optimized with both TPS and compared; 39 were for brain metastases and 20 were for vertebral metastases. To avoid bias in plan comparison, Precision plans were recomputed in RS with the dose algorithm and beam model of RS, and then compared to RS plans. The comparison was divided into 3 parts in order to reflect the potential of RS and the differences with Precision, in terms of technical aspects of delivery efficiency and dose distribution. We compared the dose to the target and to the organs at risk (OAR), the conformity index (CI), the gradient, as well as the number of monitor units (MU), and the number of beams and nodes. Finally, a global plan quality index (PQI) was calculated.

**Results:**

RS plans showed an equivalent target coverage for brain metastases but worse for vertebrae. OAR sparing was improved in RS but with a lower CI compared to Precision. Using an appropriate planning methodology in RS, plans with comparable quality to Precision could be obtained, but at the cost of a longer optimization time. The PQI obtained with RS was better than Precision, except for some brain cases.

**Conclusion:**

RS is an adequate alternative for CK planning as it is possible to obtain plan quality comparable to Precision. However, the optimization time is longer compared to Precision and more attention must be paid to the choice of the initial conditions in terms of the number of beams and nodes.

## 1 INTRODUCTION

The M6 CyberKnife (CK) (Accuray, USA) offers stereotactic treatments combining hundreds of non‐coplanar beams with various tracking methods. This makes the CK ideal for treating brain or vertebral metastases with a high dose per fraction, a limited number of fractions, and sub‐millimeter positional accuracy. In our institution, Iris, fixed and multileaf collimators (MLC) are available. The Iris collimator is designed to automatically change its aperture size during the treatment, with diameters ranging from 7.5 to 60 mm. The fixed collimator is comprised of 12 cones with diameters of 5 to 60 mm, which are manually inserted into the treatment head. It makes it possible to avoid the uncertainty of the Iris apertures and is generally used when only one diameter is enough for the treatment. Finally, a MLC is available for more irregular and/or larger targets, with a maximal aperture size of 115 × 100 mm^2^.

So far, the only available treatment planning system (TPS) for CK was Precision (Accuray, USA), in which three dose calculation algorithms are available: Raytracing for fixed and Iris collimators, finite size pencil beam^1^ (FSPB) for MLC, and Monte Carlo^2^, ^3^ (MC) for all collimators. Both RayTracing and FSPB are unsuited for use in heterogeneous conditions. In that case, the MC algorithm is used because it accounts for lateral electronic scatter and lateral electronic disequilibrium. In addition, two optimization algorithms are available with Precision: the sequential optimizer and VOLO. The superiority of the VOLO optimizer has been previously shown^4^, ^5^ and therefore, all CK plans performed in our institution are optimized with VOLO.

Recently, the TPS RayStation (RS) (RaySearch Laboratories, Sweden) introduced a module for CK planning called RayCK. RayCK offers two dose calculation algorithms: Collapsed cone^6^ (CC) and MC, available for all collimators. The CC algorithm is based on the convolution superposition method. The MC algorithm uses condensed history^7^ and calculates dose‐to‐medium, as recommended by AAPM TG 329.^8^ The modeling and validation of these algorithms were performed in the first version of RayCK (RS 11A) and have been previously presented.^9^ However, with that RS version, the optimization led to poor plan quality, especially with the Iris and fixed collimators. The poor quality of the plans was mainly due to the initial optimization conditions being too severe regarding the number of available beams that could be chosen for the optimization. As a point of comparison, in Precision the number of available beams depends on the number of collimators used, but is generally around 2000 or 3000, while in RS 11A the available beams are limited to three times the maximum number of segments, approximately 300 to 450. In version 12A, this factor, called “number of candidate beam directions”, can be manually modified and increased in the same order of magnitude as for Precision, and therefore, users can expect to achieve a better plan quality. Considering these improvements, it is now possible to compare the plan quality between Precision and RS 12A.

The purpose of the study was therefore to compare the dosimetry obtained from Precision and RS, as well as the delivery efficiency, in order to validate the use of RS TPS for CK planning without any loss of quality compared to Precision.

## 2 MATERIALS AND METHODS

### 2.1 Treatment plan calculations in Precision and RS 12A

In total, 39 plans for intracranial metastases (13 with each collimator type) and 20 plans for vertebral metastases (10 with each the Iris and the MLC) were optimized with both Precision and RS. The intracranial plans were composed of one to nine metastases and brain cavities. The MLC was used for brain cavities prescribed to 35 Gy in five fractions, with a prescription isodose of 80%. The fixed and Iris collimators were used for brain metastases. The fixed collimator was used when all lesions could be treated with the same fixed diameter. Only the 7.5‐ or the 10‐mm diameters were used. The Iris collimator was used when more than one aperture size had to be selected, with diameters between 7.5‐ and 40‐mm. Brain metastases were planned with a single fraction of 20 Gy or 24 Gy, depending on the medical diagnostic, with a prescription isodose of 70%. For vertebral plans, the planning target volume (PTV) was composed of two dose levels: a low dose (LD) of 30 Gy and a high dose (HD) of 35 Gy in five fractions with a prescription isodose of 80% for each dose level.

The optimization procedure differed between Precision and RS because of intrinsic differences in the TPS, particularly related to their respective available objective functions. In Precision these latter are the maximum and minimum doses, as well as the dose to a given volume. These functions are also available in RS, but other objective functions can be used such as the maximum equivalent uniform dose (EUD)^10^ or the dose fall‐off that behaves like a maximum dose function but with different dose levels at different distances from the target.

With both TPS the objective functions related to the PTV were a minimum dose at the prescription dose and a maximum dose corresponding to the prescribed isodose (70% or 80%). The gradient optimization was performed by reducing the dose in shells (with Precision) or rings (with RS) created around the PTV with different expansion margins. With Precision, a maximum dose was applied to each of the shells, whereas a dose fall‐off was applied in RS.

The plans have been optimized with the aim of being clinically acceptable, which implies having the highest possible PTV coverage (defined as the percentage of PTV volume receiving the prescribed dose) while respecting OARs dose constraints,^11^ which are given in Table 1. The PTV coverage objective was to obtain the prescribed dose on at least 97% of the PTV for brain cases and on at least 95% of the PTV for vertebral cases. PTV undercoverage was tolerated in order to respect dose constraints on OARs.

**TABLE 1:** Dose constraints on organs at risk for 1 and 5 fractions.

	1 fraction	5 fractions
Brain	V12Gy < 10cc	D20cc < 24 Gy
Brainstem	D0.03cc < 10 Gy	D0.03cc < 23 Gy
Chiasm	D0.03cc < 10 Gy	D0.03cc < 25 Gy
Optic nerves	D0.03cc < 10 Gy	D0.03cc < 25 Gy
Spinal cord	−^a^	D0.03cc < 25 Gy
	−	D0.25cc < 22.5 Gy
	−	D1.2cc < 20 Gy
Spinal canal	D0.03cc < 10 Gy	D0.03cc < 30 Gy
Roots	−	D0.03cc < 32 Gy
Cauda equina	−	D0.03cc < 32 Gy

^a)^ No constraints were defined.

The maximum number of nodes and beams could be limited in both TPS. The optimization of RS plans was divided into three parts: during the first part (Part 1), the RS plans were optimized with the same number of beams and nodes as allowed in Precision, as well as the same maximum and minimum MU/beam. The total number of MU was left open in both TPS. During Part 1, 1000 beam candidates and 80 iterations were used, which are the default parameters in RS when creating a CK plan. This part was meant to understand how the RS optimizer behaved compared to Precision in terms of beams, nodes, and MU. In the second part (Part 2), each RS plan was optimized by limiting the numbers of beams, nodes, and MU to the numbers actually obtained in the Precision plan, still with 1000 beam candidates and 80 iterations. This part was meant to compare the plan quality with a similar number of nodes, beams, and MU, and therefore a similar treatment delivery time. Finally, in the third part (Part 3), RS plans obtained during Part 2 were recalculated with 3000 beam candidates and 500 iterations. With this method, a large increase in the optimization time was expected, but with the aim of increasing the plan quality. The objective of Part 3 was to understand the potential of RS when removing the optimization time limitation.

To avoid inter‐ and intra‐planner variability on the optimization, each RS plan was independently optimized of the results obtained with Precision, and the same medical physicist, expert with both TPS, performed both Precision and RS plans. Additionally, to exclude the bias in plan comparison due to different dose calculation algorithms (RayTracing/FSPB/MC in Precision and CC/MC in RS), beam models (independent beam models between Precision and RS) or resolution (0.06 × 0.1 × 0.06 cm^3^ for Precision and of 0.1 × 0.1 × 0.1 cm^3^ for RS), each Precision plan was recomputed via a script in RS, keeping the same segments, beams, nodes and MU, but with the RS algorithm, beam model and a 0.1 × 0.1 × 0.1 cm^3^ resolution.

### 2.2 Comparison metrics

A gradient index (GI) defined as the ratio of the volume receiving half of the prescription dose (V50%) to the volume receiving the prescription dose (V100%)^12^ was used to compare the gradients. A lower GI indicates a steeper dose gradient. A Plan Quality Index^13^ (PQI) calculated from the conformity index (CI), defined as the ratio of the target volume covered by the prescribed dose to the total isodose volume, the dose to the target, and the dose to OARs, and adapted to stereotactic treatments, was used as a global comparison between Precision and RS plans (see Supplementary Material). The dose to the target included the PTV coverage and the maximum PTV dose. For intracranial plans, the most relevant OAR used to calculate the PQI was the brain, but other OARs such as the brainstem or optical nerves could be included if a non‐significant dose was received by the OAR. For vertebral plans, the dose to the spinal canal, spinal cord, and roots were used. The PQI could vary between 0 for a perfect plan to $\sqrt{3}$ in the worst case.

A Wilcoxon two‐sided matched‐paired signed‐rank test with a significance level of 0.05 (*p*‐value) was calculated to determine statistically significant differences for each parameter compared.

## 3 RESULTS

### 3.1 Intracranial plans

**FIGURE 1:**
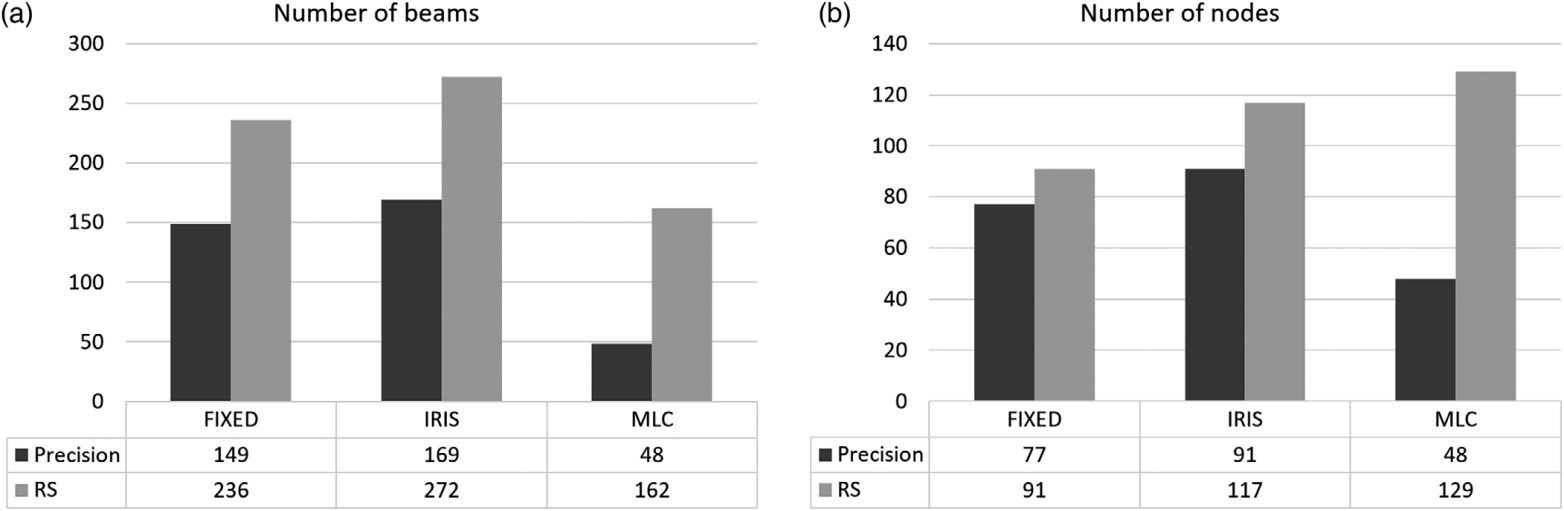
Part 1 of intracranial plans: distributions of number of beams (a) and nodes (b) for MLC, fixed, and Iris collimators for Precision (black) and RS (grey). MLC, multileaf collimator; RS, RayStation.

Figure 1 shows the differences in the number of beams and nodes for the intracranial plans with MLC, fixed, and Iris collimators obtained in Part 1. For Part 1 plans, the average number of MU with RS was increased by 13% compared to Precision for fixed and Iris collimators and by 41% for the MLC. On average, RS used 60% more beams with fixed and Iris and 238% more beams with MLC than Precision. With the MLC, RS used 375% more segments. Regarding the number of nodes, 18% more nodes were used with RS for fixed plans and up to 169% more for MLC. The only statistically non‐significant difference obtained was the number of nodes with the fixed collimator. From a clinical point of view, this increase in the number of beams, nodes, and MU led to clinically non‐acceptable plans with delivery times of over 60 min for Iris plans with more than 3 metastases.

Results for Parts 2 and 3 are shown in Table 2. During Part 2 with the fixed collimator, it was impossible to obtain clinically acceptable plans for the two plans with more than 5 metastases. Indeed, the PTV coverage obtained was 43.9% for 8 metastases and 63.3% for 9 metastases. For these two plans, given the undercoverage of the targets, the V12Gy to the brain was reduced, leading to an artificially low V12Gy for fixed plans with more than 5 metastases (see Table 2). If these two plans were removed from the analysis, and only plans with less or equal to 5 metastases were taken into account, the coverage was then comparable between Precision (97.4 ± 1.0%) and RS (97.5 ± 0.5%) and the V12Gy was lower with Precision (1.39 ± 0.90cc) than in RS (1.48 ± 0.93cc), with a *p*‐value ≤ 0.05, as seen in Table 2. By increasing the number of beam candidates and iterations (Part 3) in RS, clinically acceptable plans were obtained for the two plans with more than 5 metastases, with similar PTV coverage and lowered V12Gy than Precision plans. The same target coverage issue was not observed for plans with more than 5 metastases using the Iris collimator. When comparing Precision with RS Part 3 plans, the dose to the target (combination of the coverage and the maximal dose) was equivalent but the sparing of OAR was better with RS. The dose fall‐off outside the target, represented by the GI, was improved with RS for the fixed and Iris collimator but worse with the MLC.

**TABLE 2:** Parts 2 and 3 of intracranial plans: Results of PTV coverage, CI, V12Gy to the brain, GI, PQI, and number of nodes for Precision, RS Part 2 and Part 3 plans for all collimators.

**FIXED**		Precision	RS PART 2	RS PART 3
PTV coverage (%)	≤ 5 metastases	97.4 ± 1.0	97.5 ± 0.5	97.5 ± 0.9
	> 5 metastases	97.3 ± 1.2	53.6 ± 13.7	97.5 ± 0.7
CI	≤ 5 metastases	0.69 ± 0.08	0.67 ± 0.07	0.68 ± 0.05
	> 5 metastases	**0.68 ± 0.00**	**0.46 ± 0.07**	**0.60 ± 0.09**
V12Gy Brain (cc)	≤ 5 metastases	1.39 ± 0.90	**1.48 ± 0.93**	1.31 ± 0.79
	> 5 metastases	6.50 ± 2.69	5.58 ± 2.22	6.35 ± 2.48
GI	≤ 5 metastases	5.00 ± 1.00	5.42 ± 0.90	4.95 ± 0.67
	> 5 metastases	6.70 ± 0.71	8.14 ± 2.65	5.81 ± 0.33
Nodes	≤ 5 metastases	74	**58**	**45**
	> 5 metastases	96	123	98
PQI	≤ 5 metastases	0.36	0.38	0.36
	> 5 metastases	0.73	0.83	0.77

**IRIS**	Precision	RS PART 2	RS PART 3
PTV coverage (%)	97.0 ± 1.2	96.9 ± 0.7	97.7 ± 0.4
CI	0.81 ± 0.07	**0.76 ± 0.06**	**0.78 ± 0.05**
V12Gy Brain (cc)	10.58 ± 9.05	10.99 ± 9.53	**10.08 ± 8.81**
GI	4.33 ± 0.57	4.29 ± 0.55	**3.96 ± 0.58**
Nodes	91	**84**	**80**
PQI	0.92	0.89	0.86

**MLC**	Precision	RS PART 2	RS PART 3
PTV coverage (%)	98.27 ± 1.56	98.23 ± 0.50	98.06 ± 1.00
CI	0.88 ± 0.04	0.87 ± 0.05	0.87 ± 0.05
D20cc Brain (Gy)	21.19 ± 7.93	**22.30 ± 7.70**	21.45 ± 7.92
GI	3.11 ± 0.37	**3.61 ± 0.41**	3.73 ± 1.00
Nodes	48	42	42
PQI	0.74	0.72	**0.70**

*Note*: Results with *p* ≤ 0.05 are in bold. For the fixed collimator, the analysis is divided into plans with a number of metastases inferior or equal to 5, and superior to 5.

Abbreviations: CI, conformity index; GI, gradient index; MLC, multileaf collimator; PQI, plan quality index; PTV, planning target volume; RS, RayStation.

The PQI of RS Part 3 with the Iris and MLC was improved by 6.5% and 5.4%, respectively, compared to the Precision plans. The difference of PQI between RS Part 3 and Precision plans using the fixed collimator was 2.4%, in favor of Precision plans. The improvement of PQI from RS Part 2 to RS Part 3 plans was higher with the fixed collimator (7%) compared to Iris and MLC plans (3.5% and 2.9% respectively).

In Parts 2 and 3, the differences between Precision and RS plans in number of beams were <2%, and <5% for UM. However, for nodes, RS used on average 12% less in Part 2 and 31% less in Part 3.

### 3.2 Vertebra plans

**FIGURE 2:**
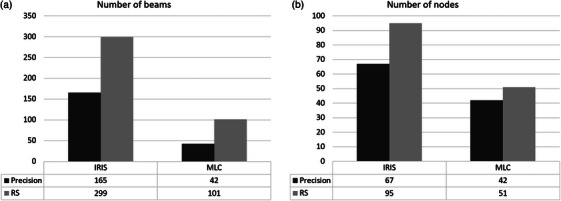
Part 1 of vertebral plans: distributions of number of beams (a) and nodes (b) for MLC and Iris collimators for Precision (black) and RS (grey). MLC, multileaf collimator; RS, RayStation.

During Part 1, the number of MU was increased with RS by 87% with Iris and 11% with MLC compared to Precision. RS MLC plans used 416% more segments than Precision. The differences in terms of the number of beams and nodes are shown in Figure 2. The only statistically non‐significant parameter was the number of MU for the MLC. From a clinical point of view, this increase in the number of beams, nodes, and MU led to clinically unacceptable plans due to the excessive delivery time.

Results for Parts 2 and 3 are shown in Table 3. For both Parts, the differences between Precision and RS plans in terms of the number of beams, segments, nodes, and MU were less than ± 5%, except for the number of nodes of MLC where the number of nodes was reduced by 49% for RS plans. For both collimators, a significant drop in PTV coverage was observed in RS plans, but the sparing of OARs was improved. In RS plans, the dose gradient was steeper with the Iris collimator but worse with the MLC. With the Iris collimator, the PQI improvement compared to Precision was 6.7% for RS Part 2 and 7.7% for RS Part 3. With MLC, the PQI of Precision and RS Part 2 plans were equal but improved by 6% with RS Part 3. For the vertebral plans that were optimized with both Iris and MLC, the PQI obtained during Part 3 showed a better PQI for Iris (0.88) than for MLC (1.01).

**TABLE 3:** Parts 2 and 3 of vertebral plans: Results of PTV coverage, CI, GI, PQI, and number of nodes for Precision, RS Part 2 and Part 3 plans for all collimators.

**IRIS**	Precision	RS PART 2	RS PART 3
PTV coverage HD/LD (%)	93.0 ± 8.0/94.8 ± 5.0	**87.3 ± 9.5/85.9 ± 6.6**	91.4 ± 7.2/**91.6 ± 3.7**
PTV CI HD/LD	0.58 ± 0.28/0.77 ± 0.14	0.59 ± 0.24/0.73 ± 0.26	0.59 ± 0.20/**0.84 ± 0.12**
GI	4.10 ± 0.61	4.08 ± 0.45	**3.74 ± 0.36**
Nodes	67	66	66
PQI	1.04	0.97	0.96

**MLC**	Precision	RS PART 2	RS PART 3
PTV coverage HD/LD (%)	97.9 ± 2.6/97.2 ± 1.3	**94.0 ± 7.4/94.0 ± 2.6**	**94.8 ± 6.3/94.6 ± 2.6**
PTV CI HD/LD	0.38 ± 0.16/0.77 ± 0.07	0.40 ± 0.13/**0.81 ± 0.09**	0.46 ± 0.16/**0.84 ± 0.09**
GI	3.56 ± 0.42	**5.22 ± 1.76**	**4.87 ± 1.34**
Nodes	41	**21**	**22**
PQI	1.06	1.06	1.00

*Note*: Results with *p* ≤ 0.05 are in bold.

Abbreviations: CI, conformity index; GI, gradient index; HD, high dose; LD, low dose; MLC, multileaf collimator; PQI, plan quality index; PTV, planning target volume; RS, RayStation.

## 4 DISCUSSION

This study compared the CK planning using Precision TPS and RS version 12A. The focus was made on brain and vertebral metastases and the overall quality of the treatment plans was compared using a global index that considered target dose, organ dose, and CI. Based on our results, RS may be used to perform CK dosimetries of brain and vertebral metastases.

### 4.1 Intracranial plans

There is a major difference in how the TPS handled the number of beams, nodes, and MU. Indeed, even when a high number of beams and nodes were allowed, Precision seemed to use only what was necessary to obtain an optimal plan. In contrast, RS used almost all the available beams and a significantly larger number of nodes and MUs, thus significantly increasing the delivery time compared to Precision. Therefore, when starting an RS plan, the planner should limit the maximum authorized number of beams and nodes. Hence, a good knowledge and expertise of the optimal number of nodes and beams necessary for a given plan configuration (e.g., number of metastases) is required.

When optimizing plans of more than 5 metastases in RS with the fixed collimator with 1000 beam candidates (RS Part 2), the optimization led to non‐clinically acceptable plans. However, by increasing the number of beam candidates to 3000, as well as the number of iterations, a quality equivalent to the Precision plans was achieved. The counterpart was the longer optimization time, which significantly increased as the number of beam candidates and iterations increased. To give an estimate, by going from 1000 to 3000 beam candidates, keeping the same number of iterations, the optimization time was increased by a factor of around 2.5, increasing optimization time for a simple brain case from 145 to 355 s for 80 iterations. If the number of iterations was increased to 500, with 3000 beam candidates (Part 3), the optimization time was 560 s, which is almost 4 times longer than with 1000 beam candidates and 80 iterations (Part 2). If for the fixed collimator this increase in optimization time is mandatory to obtain clinically acceptable plans, for Iris and MLC, clinically acceptable plans were already obtained during Part 2, and the maximum PQI difference between RS Parts 2 and 3 was 3.5%. Therefore, the increase in plan quality must be weighed against the large increase in calculation time.

For the fixed collimator, only a small difference in PQI was obtained between RS Part 3 and Precision plans, thus indicating a very similar overall plan quality. However, for Iris and MLC collimators, larger differences in PQI were obtained, indicating an increase in plan quality with RS, mainly due to a better sparing of OARs.

For Iris and fixed plans, steeper gradients were obtained with RS, which explains the dose decrease to the brain. In contrast, with MLC, steeper gradients were obtained with Precision, and the dose to the brain was slightly lower than in RS plans. For MLC plans, target volumes being larger, the chiasma, the brainstem, and the optic nerves each received a significant dose and were therefore included in the PQI calculations. For these organs, the dose was significantly reduced with RS plans, which explains the better PQI obtained with RS plans.

For fixed collimator plans with less than 5 metastases and for Iris collimator, considering a similar or better PQI for RS Part 3 plans compared to Precision, as well as a lower V12Gy brain dose and a better gradient for RS plans, RS is a suitable alternative to Precision, if plans are calculated following the Part 3 methodology. For a fixed collimator with more than 5 metastases, although the gradient and the V12Gy were improved in RS Part 3, the PQI was worse due to a decrease in CI. However, plans obtained with RS were clinically acceptable. For MLC, although the PQI was better for RS Part 3 plans, the brain dose and the gradient were not as good, so the superiority of RS over Precision is not guaranteed. However, the plans obtained were clinically acceptable, with no major loss of quality compared to Precision plans.

### 4.2 Vertebra plans

The distributions of the number of beams, nodes, and MUs led to the same conclusion as for intracranial plans.

Although the PQI of RS plans was similar to or better than Precision, thanks to a better sparing of OARs with RS, the decrease in PTV coverage was particularly concerning for RS plans for both collimators. Further studies would be needed to ensure that with an equivalent dose to OARs, an at least equivalent dose to the targets could be obtained with RS. When comparing RS Part 3 plans performed with both the Iris and MLC collimators, the PQI of the Iris plans was better due to a better CI and sparing of OARs than in MLC plans. Iris plans also showed steeper gradients than MLC plans. Despite this, it appeared that the loss of coverage was less significant with the MLC. Therefore, MLC should be preferred for vertebra planning over Iris. When optimizing with the MLC, RS used half the authorized number of nodes (so half the number of nodes of Precision plans), but with a similar number of beams, segments, and MU. Therefore, the average number of segments per beam was the same, but with an increased number of beams per node. One can imagine that with an increased number of nodes, even with the same number of beams, steeper gradients could be obtained, as well as better coverage. By slightly relaxing the dose to the OARs and by increasing the number of beams allowed, a similar target coverage than Precision plans could probably be obtained, without an increase in delivery time. This should be investigated in further studies.

The increase of optimization time due to the increased number of beam candidates and iterations was beneficial, but not mandatory, to obtain clinically acceptable plans with the MLC. For the Iris collimator, the increase in plan quality between RS Part 2 and Part 3 was insignificant.

### 4.3 Limitations

There were some limitations to our study. First, even though we tried to use the most homogeneous and reproducible planning method to optimize all the plans, the optimization was made manually and over several weeks, which can lead to a certain inhomogeneity. However, some of this inhomogeneity should be mitigated by the number of plans compared. In addition, the CK plans in RS were done directly after the RS 12A was installed so it seems reasonable to say there is room for improvement in CK planning with RS, notably with the fixed and Iris collimators which had not been used before the RS 12A was installed. Secondly, the PQI used to compare the plans was only based on the dose to the targets and OARs and on the CI. However, other parameters are also important for dosimetric evaluation, such as the dose gradient or the dose distribution. This index therefore gave only a partial idea of the plan quality.

## 5 CONCLUSION

Our study demonstrated that RS 12A could be used clinically for CK planning of cerebral and vertebral metastases, with some limited adaptations to be done compared to Precision planning methodology. For brain plans with the fixed collimator, to obtain a similar plan quality as Precision, the optimization time must be increased, which can be a potential limitation in a clinical rush. In addition, good experience in planning CK plans is necessary in order to obtain plans with an acceptable delivery time. For other collimators, a similar plan quality was obtained with an acceptable optimization time. For vertebral metastases, despite a similar or better PQI for RS plans, a significant decrease in coverage was observed and should be further investigated. The use of MLC for RS plans made it possible to minimize the loss of coverage compared to Iris and therefore the MLC should be preferred for the planning of vertebrae.

## AUTHOR CONTRIBUTIONS

**Maude Gondré**: Conceptualization; data curation; formal analysis; investigation; methodology; resources; software; writing—original draft; writing—review & editing. **Véronique Vallet**: Conceptualization; methodology; supervision; validation; writing—review & editing. **Jean Bourhis**: Validation; writing—review & editing. **François Bochud**: Conceptualization; validation; writing—review & editing. **Raphaël Moeckli**: Conceptualization; methodology; project administration; supervision; validation; writing—original draft; writing—review & editing.

## CONFLICT OF INTEREST STATEMENT

The authors declare no conflicts of interest.

## Supporting information



Supporting Information
