# Supplementary material for: Dosimetric comparison of M6 CyberKnife plans optimized with Precision and RayStation 12A treatment planning systems
Source: J Appl Clin Med Phys. 2024 Dec 19;26(3):e14585. doi: 10.1002/acm2.14585 (PMC11905244; doi:10.1002/acm2.14585)
Supplement: Supplementary file 1 — Supporting Information [file ACM2-26-e14585-s001.docx]

**SUPPLEMENTARY MATERIAL**

A Plan Quality Index (PQI) was used as a global comparison between Precision and RS plans. This index was calculated using three parameters: the conformity index (CI), the dose to the target (denoted by M hereafter), and the dose to the OAR (denoted by P hereafter). The CI was defined as the ratio of the target volume covered by the prescribed dose to the total isodose volume. The dose to the target depended on two parameters: the coverage, defined as the volume of the PTV receiving the prescription dose, and the maximum dose inside the PTV. The dose to the OAR was compared to checkpoints[13] defined in Table 1. The coverage criterion for cerebral cases was that at least 97% of the PTV should receive the prescribed dose. For vertebral cases, the criterion was that at least 95% of the PTV should receive the prescribed dose. Regarding the maximum dose, the criterion was a maximum deviation to the desired prescription isodose of ±3%. The PQI was then given by:

$$PQI= \sqrt{\left( 1-M \right)^{2}+\left( 1-P \right)^{2}+\left( 1-CI \right)^{2}}$$

Where the definitions of M and P give:

$$\mathrm{PQI}= \sqrt{\left( 1-\frac{\frac{V_{PTV,100\%}}{V_{PTV criteria}}+\left( 1-\frac{\left| \Delta_{\mathrm{isodose}} \right|}{3\%} \right)}{\frac{100}{V_{PTV criteria}}+1} \right)^{2}+\left( 1-\frac{1}{n}\sum_{j=1}^{n} \left\{ \frac{1}{m}\sum_{i=1}^{m} 1-\frac{V_{OAR,dose}}{V_{OAR,dose limit}} \right\} \right)^{2}+\left( 1-CI \right)^{2}}$$

With V_PTV,100%_ the PTV volume receiving at least 100% of the prescribed dose. For vertebral cases with two dose levels, this factor was adapted to$\frac{2}{3}V_{PTV HD,100\%}+\frac{1}{3}V_{PTV LD,100\%}$, allowing for a greater weight on the HD PTV than on the LD PTV. $V_{PTV criteria}$was the criteria in term of PTV coverage. Δ_isodose_ was the difference between the obtained and desired isodose, $n$ the number of OAR evaluated, $m$ the number of checkpoints for each OAR, V_OAR,dose limit_ the constraint applied on the OAR and V_OAR,dose_, the dose obtained for that specific constraint. For vertebra, the CI was defined as $\frac{1}{2}\left( CI_{\mathrm{LD}}+CI_{\mathrm{HD}} \right)$.
